# Chinese herbal medicine Wutou decoction for knee osteoarthritis: A protocol for systematic review and meta-analysis

**DOI:** 10.1097/MD.0000000000022767

**Published:** 2020-10-23

**Authors:** Xing Zhou, Kemeng Xiang, Xiangyao Yuan, Zhenping Wang, Kuanglin Li

**Affiliations:** Taizhou Traditional Chinese Medicine Hospital, Zhejiang Province, China.

**Keywords:** knee osteoarthritis, protocol, randomized controlled trial, systematic review, Wutou decoction

## Abstract

**Background:**

Knee osteoarthritis (KOA) causes joint pain and limited mobility, which affects the quality of life. The use of Chinese herbal medicine to treat KOA has achieved certain effects, and Wutou decoction (WTD) is one of them. But there is no high-level evidence to support this result. The purpose of this work is to evaluate WTD's efficacy and safety in the management of KOA.

**Methods:**

We will search articles in 7 electronic databases including Chinese National Knowledge Infrastructure (CNKI), Wanfang Data (WF), Chinese Scientific Journals Database (VIP), Chinese databases SinoMed (CBM), PubMed, Embase, and Cochrane Library databases. All the publications, with no time restrictions, will be searched without any restriction of language and status, the time from the establishment of the database to September 2020. Two reviewers will independently assess the quality of the selected studies, NoteExpress and Excel software will be used to extract data, and the content will be stored in an electronic chart. Different researchers will separately screen the titles and abstracts of records acquired potential eligibility which comes from the electronic databases. Full-text screening and data extraction will be conducted afterward independently. Statistical analysis will be conducted using RevMan 5.4 software.

**Results:**

This study will evaluate the current efficacy and safety of WTD in the treatment of KOA, to provide high-quality, evidence-based clinical recommendations.

**Conclusion:**

This study will provide reliable evidence on whether WTD is safe and effective in treating KOA.

**Trial registration number:**

INPLASY202090022

## 1 Introduction

The number of patients with osteoarthritis worldwide will continue to increase in the coming decades, and it will become one of the most common diseases.^[1]^ Among patients with osteoarthritis, knee joints are the most common,^[2,3]^ and the prevalence is higher in the middle-aged and elderly population, among which women are higher than men.^[1]^ The reported incidence of knee osteoarthritis (KOA) with radiographic case definition ranges from 7.1% to –70.8%.^[4,5]^ KOA has caused a huge burden on the patients themselves and the society, accounting for about 85% of the global burden of osteoarthritis.^[6]^ Therefore, there is an urgent thing to seek safe and effective ways to relieve pain and promote the functions of patients, improve the quality of life, and reduce personal and social burdens.

The pathological changes of KOA involve articular cartilage, subchondral bone, synovium, joint capsule, and surrounding muscles and ligaments.^[7,8]^ KOA is a kind of degenerative disease, which is closely related to aging, but it is not entirely caused by aging, but more of its disease.^[9]^ Because matrix metalloproteinases (MMP) or degradative enzymes are overexpressed in the development of osteoarthritis, the balance is disrupted, resulting in a decrease in the number of proteoglycans, and ultimately causing damage to articular cartilage.^[10]^ Clinically, it is manifested as joint pain and loss of function.^[11]^

In the management and treatment of KOA, it is mainly divided into surgical intervention and conservative treatment.^[12]^ The first choice of drug therapy in conservative treatment is nonsteroidal anti-inflammatory drugs.^[12]^ The American Academy of Orthopedic Surgeons (AAOS) guidelines have evidence-based medical evidence to support this choice. However, long-term use of nonsteroidal anti-inflammatory drugs may carry a higher long-term risk of more serious complications.^[13]^

Chinese medicine displayed promoted results over other methods for treating KOA. Chinese herbal medicine has multiple types and multiple ways of intervention, including fumigation, iontophoresis, hot compressed, etc.^[14]^ Traditional Chinese medicine (TCM) is effective in treating symptomatic KOA,^[16]^ and has better efficacy and safety than some western medicines.^[15,17]^

Wutou decoction (WTD) is derived from Zhang Zhongjing, a famous Chinese medicine practitioner, It is composed of 5 TCMs: Radix Aconiti, Herba Ephedra, Radix Astragali, Radix Paeoniae Alba, and Radix Glycytthizae.^[18]^ That is widely used to treat arthritis and pain of joints,^[19,20]^ Animal experiments show that, WTD may modulate DNA methylation and histone modifications, has anti-inflammatory effects on a collagen-induced arthritis rat model.^[21]^ Through network pharmacology research, it is found that WTD can treat osteoarthritis mainly by interfering with cell cycle, inflammation, and endocrine pathways.^[22]^ However, currently, there is no higher-level evidence-based medical evidence to systematically evaluate and analyze the safety and efficacy of WTD in the treatment of KOA. Therefore, this work aims to achieve the above-mentioned goals through systematic reviews and meta-analysis, and provide reliable evidence for the clinic.

## 2 Methods

### 2.1 Study registration

This protocol report is structured according to the Preferred Reporting Items for Systematic Reviews and Meta-analysis Protocols (PRISMA-P) statement.^[23]^ It is registered on the International Prospective Register of Systematic Reviews. (Registration number INPLASY202090022; https://inplasy.com/inplasy-2020-9-0022/.)

### 2.2 Inclusion criteria

#### 2.2.1 Type of study

Only randomized controlled trials (RCTs) will be included irrespective of blinding, publication status, or language in this study.

#### 2.2.2 Types of participants

Patients were diagnosed with KOA and the study belongs to a RCT. Clinical results included clinical effectiveness and visual analog scale. The experimental group must contain WTD or modified WTD and the control group was not limited except that. Otherwise, studies will be excluded if they cannot meet the inclusion criteria.

#### 2.2.3 Types of interventions

Interventions of the experimental group are WTD or modified WTD. There are no restrictions on the way of administration, dosage, and treatment period.

#### 2.2.4 Types of control groups

The control group has other treatment methods different from WTD or modified WTD.

#### 2.2.5 Outcomes

##### 2.2.5.1 Primary outcome measures

The primary outcome is visual analog scale.^[24]^

##### 2.2.5.2 Secondary outcomes

The secondary outcomes are clinical effectiveness and the incidence of adverse reactions.

### 2.3 Search strategy

CNKI, Wanfang, VIP, CBM, PubMed, Embase, and Cochrane Library databases were searched for this study. Take the subject terms combined with free words to search, take PubMed as an example: terms consist of disease (osteoarthritis, knee OR knee osteoarthritis OR knee osteoarthritis OR Osteoarthritis of Knee OR Osteoarthritis of the Knee) and intervention (Wutou decoction OR Wutou Tang OR modified Wutou decoction) and research types (RCT OR controlled clinical trial OR random trials) as shown in Table 1.

**Table 1:**
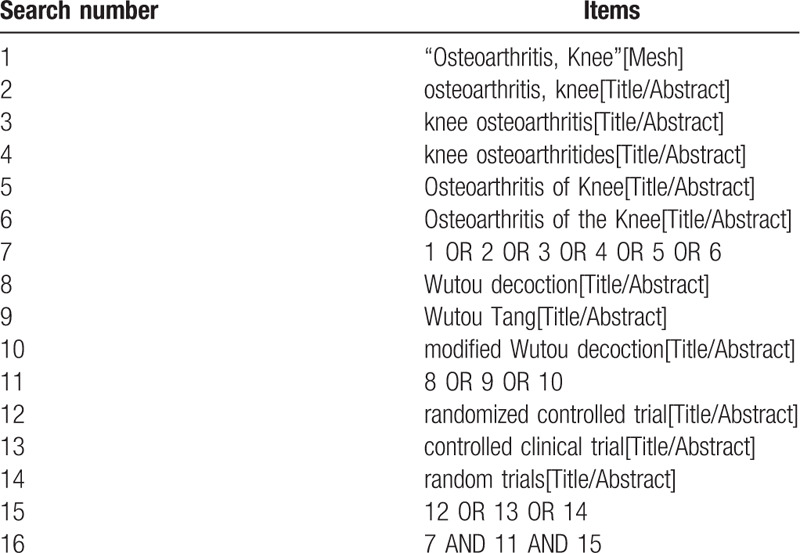
Pubmed database search strategy.

### 2.4 Data collection and analysis

#### 2.4.1 Selection of studies

**Figure 1:**
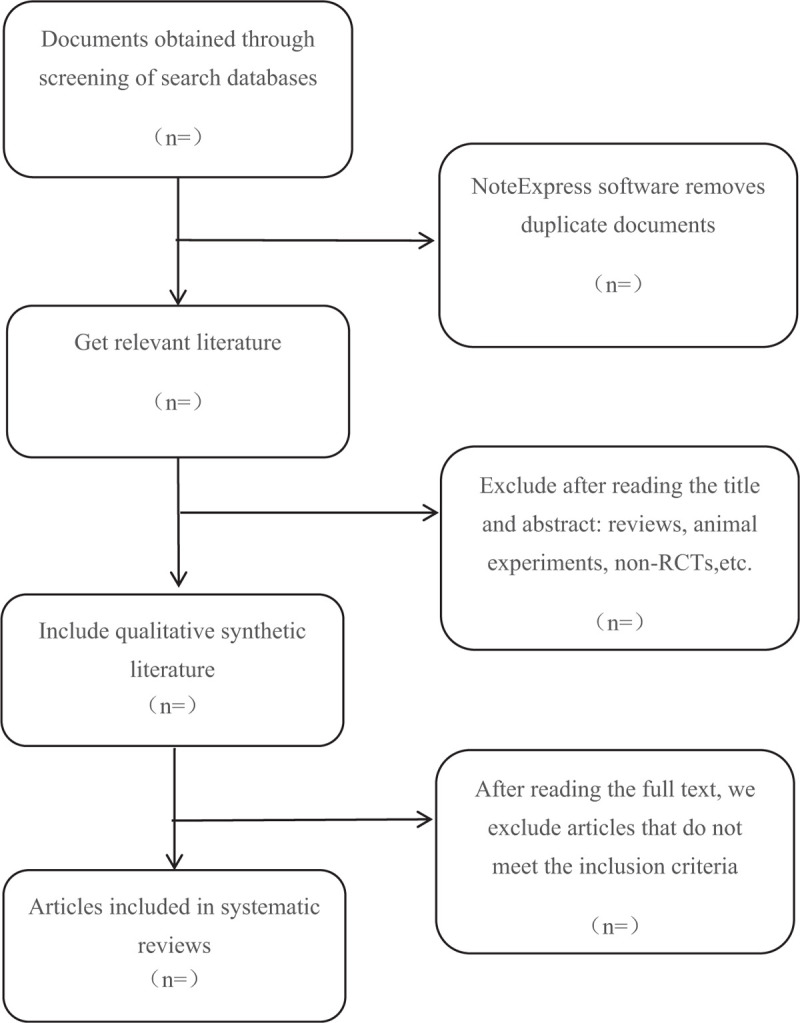
Flowchart of literature selection.

Different researchers will separately screen the titles and abstracts of records acquired potential eligibility which comes from the electronic databases. The obtained literature is managed by Notoexpress, irrelevant and duplicate articles are excluded by reading the title and abstract. Full texts screening and data extraction will be conducted afterward independently, and finally selected according to the inclusion criteria. Any disagreement will be resolved by discussion until consensus is reached or by consulting a third author. Preferred Reporting Items for Systematic Reviews and Meta-analysis Protocols (PRISMA-P) flowchart will be used to show the selection procedure (Fig. 1).

#### 2.4.2 Data extraction and management

NoteExpress and Excel software will be used to extract data, and the content will be stored in an electronic chart. The following data will be extracted: author, year of publication, country, interventions of experimental groups and control groups, time point, outcome measures, age of patients, the total number of people included in the study, patients’ basic information, etc. Different researchers will separately extract data. Any disagreement regarding data extraction will be resolved by discussion until consensus is reached or by consulting a third author.

### 2.5 Risk of bias assessment

Two reviewers will independently assess the quality of the selected studies according to the Cochrane Collaboration's tool for RCTs.^[25]^ Items will be evaluated in 3 categories: Low risk of bias, unclear bias, and high risk of bias. The following characteristics will be evaluated: random sequence generation (selection bias), allocation concealment (selection bias), blinding of participants and personnel (performance bias), incomplete outcome data (attrition bias), selective reporting (reporting bias), and other biases. Results from these questions will be graphed and assessed using Review Manager 5.4. The results will be presented in the form of a graph and will be independently evaluated by 2 researchers. If there are differences of opinion, they will be discussed with the third researcher

### 2.6 Statistical analysis

Statistical analysis will be conducted using RevMan 5.4 software. For continuous data, will be used mean difference as the effect indicator with 95% confidence interval, and dichotomous data will be calculated as risk ratio or odds ratio as the effect index with 95% confidence interval. The *I*^2^ statistic will be used to assess levels of the heterogeneity, when *I*^2^ < 50%, the fixed-effect model can be used for analysis, otherwise, the random-effect model will be used.

### 2.7 Sensitivity analysis

Through sensitivity analysis assess the source of heterogeneity, by excluding low-quality studies, or by excluding one of the included studies in turn, if there is no significant change in the heterogeneity, the results are robust, otherwise, the excluded study may be the heterogeneous originate.

### 2.8 Subgroup analysis

We will consider the subgroup analysis intervention of the experimental group.

### 2.9 Publication bias

In this study, less than ten RCTs will use funnel plots to evaluate publication bias, or else, Egger regression test will be used.

## 3 Discussion

TCM has accumulated a lot of experience in the treatment of joint pain and arthritis. The use of Chinese herbal medicine to treat KOA has also achieved good results. WTD has a good anti-inflammatory effect, which can relieve knee joint pain, and its curative effect has been confirmed clinically. However, there is still a lack of higher-level evidence-based medicine evidence to support this choice. Therefore, this study is to provide a more credible basis for future clinicians to make decisions.

This study still has certain shortcomings, because some factors will lead to biased results, such as low-quality original research, the intervention period, etc, which will weaken the reliability of the evidence.

## Author contributions

**Conceptualization:** Zhou Xing, Xiang Kemeng.

**Data curation:** Zhou Xing, Xiang Kemeng, Yuan Xiangyao, Wang Zhenping, Li Kuanglin.

**Formal analysis:** Yuan Xiangyao, Li Kuanglin.

**Investigation:** Wang Zhenping, Li Kuanglin.

**Methodology:** Zhou Xing, Xiang Kemeng, Li Kuanglin.

**Software:** Xiang Kemeng, Wang Zhenping.

**Supervision:** Yuan Xiangyao, Wang Zhenping, Li Kuanglin.

**Writing – original draft:** Zhou Xing, Xiang Kemeng.

**Writing – review & editing:** Yuan Xiangyao, Wang Zhenping.
